# (Cu_2_O-Au) – Graphene - Au layered structures as efficient near Infra - Red SERS substrates

**DOI:** 10.1038/s41598-020-60874-x

**Published:** 2020-03-05

**Authors:** Radhika V. Nair, V. M. Murukeshan

**Affiliations:** Center for Optical and Laser Engineering (COLE), School of Mechanical and Aerospace Engineering, Nanyang Technological University (NTU), Singapore, 639798 Singapore

**Keywords:** Optical properties and devices, Nanoparticles

## Abstract

Near Infra-Red Surface Enhanced Raman Spectroscopy (NIR SERS) has gained huge attention in recent years as the conventional visible SERS suffers from overwhelming fluorescence background from the fluorophore resulting in the masking of Raman signals. In this paper, we propose a novel multi-layered SERS substrate- (Cu_2_O - Au) - Graphene – Au - for efficient NIR SERS applications. The proposed structure has a monolayer of Cu_2_O - Au core-shell particles on a Au substrate with 1 nm thick graphene spacer layer. Mie simulations are used to optimize the aspect ratios of core-shell particles to shift their plasmon resonances to NIR region using MieLab software. Further, Finite Difference Time Domain (FDTD) simulations using Lumerical software are used for the design of the multiparticle layered SERS substrate as MieLab software works only for single particle systems. Designed structure is shown to provide high field enhancement factor of the order of 10^8^ at an excitation of 1064 nm thus ensuring the possibility of using the proposed structure as efficient NIR SERS substrate which could probably be used for various NIR sensing applications.

## Introduction

Rapid growth in biosensing and bioimaging research fields in the recent past involved potential development of novel nanostructured materials and various imaging techniques that involved optical probes, specialty fiber optics, Surface Enhanced Raman Spectroscopy (SERS)^1–5^. Out of these, SERS was used in the trace analysis of various chemicals, finding potential applications in fields such as biochemistry, medical diagonostics, food contamination detection, water safety, trace level explosive detection and forensics^6^. Raman signals of the analyte molecules get enhanced in SERS by the amplification of light due to the generation of localized surface plasmons in the gaps and sharp edges of metallic nanoparticles, micro or nanopatterned structures or on the roughened metallic surfaces^7–10^. Large enhancement in Raman signals is achieved when the plasmon resonance wavelength almost matches with the excitation wavelength which generally falls within the visible to NIR region (400 to 850 nm)^11^. Visible light excitation in SERS may result in photobleaching, plasmonic heating and also in an overwhelming fluorescence background as most of the fluorophore analytes have strong emission within the visible region resulting in the masking of Raman signals^12^. These problems can be overcome by using larger wavelength excitations (NIR lasers)^13^. Tunability in plasmon resonances to match with the exciation wavelength can be achieved by the modification of metallic or metal hybrid nanoparticle morphologies or using core-shell particles^14^. Different metal-dielectric, dielectric-metal, metal-metal and multilayer metal-dielectric structures are shown to have large tunability of plasmon resonances from visible to NIR region^15,16^. Various imaging techniques can be used for nanoscale level imaging of such structures^17,18^. Plasmonic resonances of core-shell particles are highly sensitive to core radius, shell thickess and refractive indices of core and shell^19^. High refractive index dielectric core enables efficient coupling between spherical and cavity plasmons leading to large tunability of symmetric modes (longer wavelength modes)^20^. Cu_2_O is a high refractive index semiconductor (~2.6 in NIR) which along with metallic shell provides larger visible to NIR tunability in plasmonic resonances compared to other semiconductors^21^. Current interest in the design of SERS substrates is in metal- dielectric-metal multilayer structures with a thin graphene spacer layer which enables efficient coupling of plasmons with the metallic substrates^22^.

The aim of our present work is to model a novel and efficient NIR SERS substrate with large SERS field enhancement factor values at NIR excitation wavelength of 1064 nm. In general, fluorophores used in biological applications - Thioflavin, Cyanine dyes etc. have strong emission within 400 nm to 750 nm wavelength range^23,24^. Longer excitation wavelength of 1064 nm is hence chosen to avoid the overwhelming fluorescence background from fluorophores. SERS enhancement in graphene monolayer and NIR SERS by hollow Au nanotags are proposed earlier^25,26^. Plasmon resonances below 800 nm are made use of in the above works which is far below the excitation wavelength 1064 nm. Here we propose a layered NIR SERS substrate with monolayer of Cu_2_O - Au core-shell particles on Au substrate separated by 1 nm thick graphene layer. Layered structure is designed in such a way that the plasmon resonance exactly matches with 1064 nm which can provide enhanced NIR SERS properties. Mie computations and FDTD simulations are used to explore the potential of the proposed structure in NIR SERS applications. Mie calculations using MieLab software is used for the plasmon resonance study of single core-shell particle systems. MieLab software can only be used for the study of single particle systems. Due to this limitation, further modelling of multi core-shell particle embedded SERS substrate is carried out using Lumerical FDTD simulations. Mie theory used for the study of absorption efficiencies of core-shell particles is discussed in the next section.

## Absorption Efficiency of Cu_2_O - Au Core-Shell Particles

Mie theory in the modified form for core-shell particles is used for the calculation of the absorption efficiency of Cu_2_O – Au core-shell particles in the near infra-red region^27^. According to Mie theory,

1 $${{\rm{Q}}}_{{\rm{sca}}}=\frac{2}{{z}^{2}}{\sum }_{l=1}^{\infty }(2l+1)({|{a}_{l}|}^{2}+{|{b}_{l}|}^{2})$$

2 $${{\rm{Q}}}_{{\rm{ext}}}=\,\frac{2}{{z}^{2}}\,{Re}{\sum }_{{\rm{l}}=1}^{\infty }(2{\rm{l}}+1)({{\rm{a}}}_{{\rm{l}}}+{{\rm{b}}}_{{\rm{l}}})$$

3 $${{\rm{Q}}}_{{\rm{abs}}}={{\rm{Q}}}_{{\rm{ext}}}-{{\rm{Q}}}_{{\rm{sca}}}$$

where *Q*_*sca*_*, Q*_*abs*_ and *Q*_*ext*_ are the scattering, absorption and extinction efficiencies of particles, z is the size parameter *=* $\frac{2\pi Rm}{\lambda }$*, R* is the radius of the particle, *λ* is the wavelength of light, $m=\frac{{n}_{s}}{{n}_{m}}$ where *n*_*s*_ is the refractive index of the spherical particle and *n*_*m*_ is the refractive index of the medium and *a*_*l*_ and *b*_*l*_ are the Mie coefficients.

For core-shell particles (core radius *a* and outer radius *b*) with core refractive index *m*_1_ and shell refractive index *m*_2_, Mie coefficients *a*_*l*_ and *b*_*l*_ are calculated using the modified relations^21^,

4 $${a}_{l}=\frac{{\psi }_{l}(y)[{\psi }_{l}^{\text{'}}({m}_{2}y)-{D}_{l}{\zeta }_{l}^{\text{'}}({m}_{2}y)]-{\psi }_{l}^{\text{'}}(y)[{\psi }_{l}({m}_{2}y)-{D}_{l}{\zeta }_{l}({m}_{2}y)]}{{\chi }_{l}(y)[{\psi }_{l}^{\text{'}}({m}_{2}y)-{D}_{l}{\zeta }_{l}^{\text{'}}({m}_{2}y)]-{\chi }_{l}^{\text{'}}(y)[{\psi }_{l}({m}_{2}y)-{D}_{l}{\zeta }_{l}({m}_{2}y)]}$$

5 $${b}_{l}=\frac{{m}_{2}{\psi }_{l}(y)[{\psi }_{l}^{\text{'}}({m}_{2}y)-{G}_{l}{\zeta }_{l}^{\text{'}}({m}_{2}y)]-{\psi }_{l}^{\text{'}}(y)[{\psi }_{l}({m}_{2}y)-{G}_{l}{\zeta }_{l}({m}_{2}y)]}{{m}_{2}{\chi }_{l}(y)[{\psi }_{l}^{\text{'}}({m}_{2}y)-{G}_{l}{\zeta }_{l}^{\text{'}}({m}_{2}y)]-{\chi }_{l}^{\text{'}}(y)[{\psi }_{l}({m}_{2}y)-{G}_{l}{\zeta }_{l}({m}_{2}y)]}$$

where,

6 $${{\rm{D}}}_{{\rm{l}}}=\frac{{m}_{2}{\psi }_{l}({m}_{2}x){\psi }_{l}^{\text{'}}({m}_{1}x)-{m}_{1}{\psi }_{l}^{\text{'}}({m}_{2}x){\psi }_{l}({m}_{1}x)}{{m}_{2}{\zeta }_{l}({m}_{2}x){\psi }_{l}^{\text{'}}({m}_{1}x)-{m}_{1}{\zeta }_{l}^{\text{'}}({m}_{2}x){\psi }_{l}({m}_{1}x)}$$

7 $${{\rm{G}}}_{{\rm{l}}}=\frac{{m}_{2}{\psi }_{l}({m}_{1}x){\psi }_{l}^{\text{'}}({m}_{2}x)-{m}_{1}{\psi }_{l}^{\text{'}}({m}_{1}x){\psi }_{l}({m}_{2}x)}{{m}_{2}{\psi }_{l}({m}_{1}x){\zeta }_{l}^{\text{'}}({m}_{2}x)-{m}_{1}{\psi }_{l}^{\text{'}}({m}_{1}x){\zeta }_{l}({m}_{2}x)}$$

For each angular momentum *l*, the shift in the plasmon resonances of core-shell particles is in accordance with the relation^15^,

8 $${\omega }_{l\pm }^{2}=\frac{{\omega }_{p}^{2}}{2}[1\pm \frac{1}{(2l+1)}\sqrt{1+4l(l+1){(\frac{a}{b})}^{2l+1}]}$$

${\omega }_{l+}\,and\,{\omega }_{l-}$ represent the frequencies of antisymmetric and symmetric modes, ${\omega }_{p}$ is the bulk plasma frequency and a and b are the inner and outer radii of the shell^28^.

**Figure 1:**
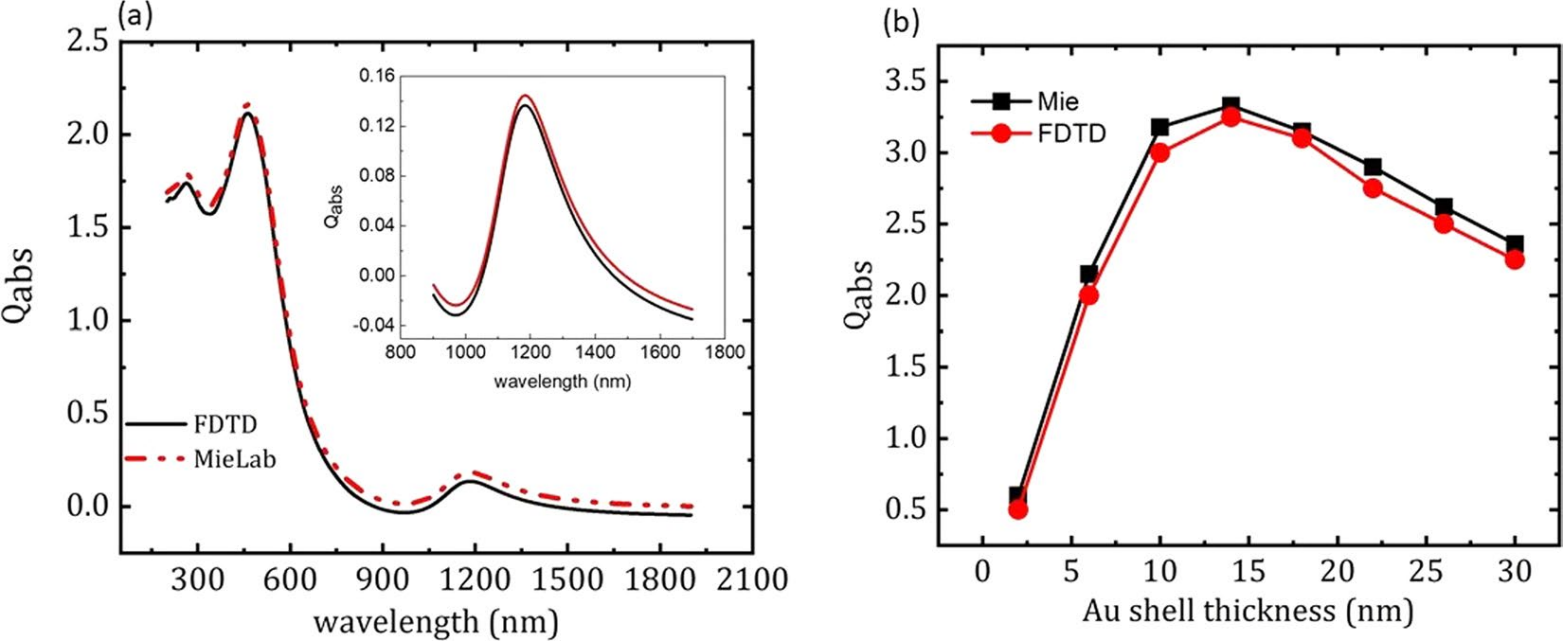
(**a**) Comparison between Q_abs_ values of Cu_2_O (60 nm)-Au (10 nm) core-shell particle calculated using FDTD and MieLab softwares. Inset shows comparison of Q_abs_ values for Cu_2_O (50 nm)-Au core-shell particles. (**b**) variation in Mie and FDTD calculations for the entire Au shell thickness values.

Q_abs_ values of Cu_2_O - Au core-shell particles in the near infra-red region are calculated using MieLab software. MieLab is a dedicated software to calculate the absorption and scattering properties single core-shell particles. FDTD can also be used for similar studies without any discrepancy of the results. Figure 1(a) shows the comparison of absorption efficiency calculations using MieLab and lumerical FDTD softwares. Absorption efficiency is calculated for Cu_2_O - Au core-shell particle with core radius 60 nm and shell thickness 10 nm using both MieLab and lumerical FDTD softwares. It is observed that there is no discernible disparity in the results obtained using both the softwares. The peak values are observed to be the same in both the simulations. Q_abs_ value obtained using FDTD simulation (0.14) is found to be comparable with the value obtained using MieLab simulation (0.15). Figure 1(b) shows the variation in Mie and FDTD calculations for the entire Au shell thickness values.

For the ease of simulation we have used MieLab for single particle studies. It is not possible to use MieLab for multiparticle systems. Hence we have used FDTD for further modelling of layered substrates and visualization of electric field. Refractive index data required for simulation for Au and Cu_2_O are obtained from Johnson and Christy data^29^ and Querry *et al*.^30^ respectively. We have performed Mie calculations on various aspect ratios of Cu_2_O-Au core-shell particles. Table 1 shows plasmon resonance positions obtained for Cu_2_O-Au core-shell particles with core radius 40 nm, 50 nm and 60 nm and with Au shell thickness in each case varying from 2 nm to 30 nm in steps of 4 nm. Plasmon resonance tunability with increase in Au shell thickness for Cu_2_O (40 nm)-Au, Cu_2_O (50 nm)-Au and Cu_2_O (60 nm) –Au is observed to be from 1325 nm to 505 nm, 1500 nm to 675 nm and 1630 nm to 712 nm, respectively.

**Table 1:** Plasmon resonance tunability data of Cu_2_O - Au core-shell particles with different core radii and Au shell thicknesses.

Au shell thickness	λ (nm)		
(nm)	Cu_2_O (40 nm)-Au	Cu_2_O (50 nm)-Au	Cu_2_O (60 nm)-Au
2	1325	1500	1630
6	815	1040	1100
10	630	850	905
14	585	775	830
18	550	735	784
22	535	710	750
26	520	690	732
30	505	675	712

**Figure 2:**
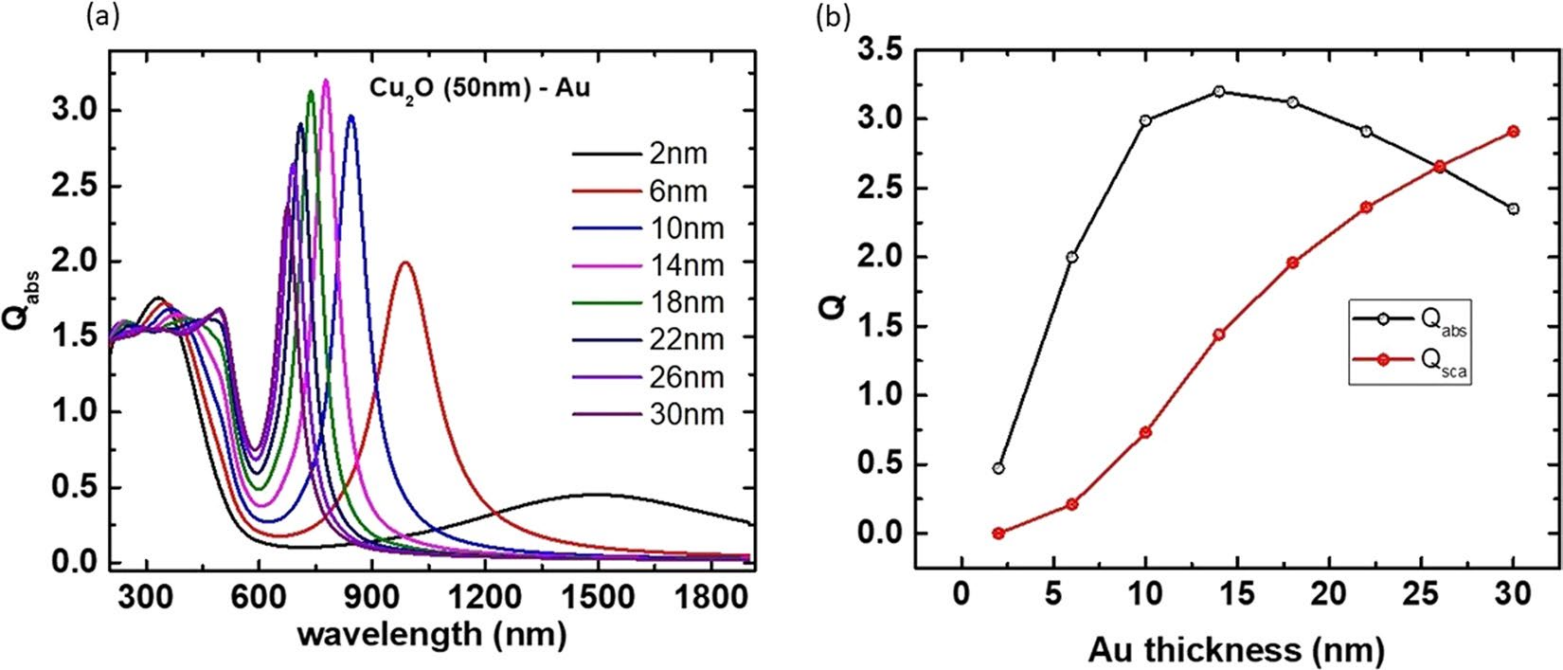
(**a**) Absorption efficiency vs. wavelength plots of Cu_2_O (50 nm)-Au core-shell particles with different Au shell thicknesses, (**b**) Absorption and scattering efficiency vs. shell thickness plots for Cu_2_O (50 nm) - Au core-shell particles. Yellow arrow indicates the Q_abs_ value obtained for Cu_2_O (50 nm) –Au (6 nm) at the resonance wavelength of 1040 nm which is very close to the desired excitation of 1064 nm.

Among the different aspect ratios, Cu_2_O - Au core-shell particle with core radius 50 nm and Au shell thickness (2 nm to 30 nm) are found to have tunable plasmonic resonance within the desired biological window of 650 nm to 1350 nm (Fig. 2(a)) and the corresponding Q_abs_ and Q_sca_ values are shown in Fig. 2(b). From Fig. 2(a) it can be inferred that the plasmon resonances of particles get blue shifted with increase in shell thickness (Eq. 8). For Cu_2_O (50 nm) - Au core-shell particles, plasmon resonances are found to be tunable from 675 nm to 1500 nm which falls within the desired NIR biological window. Absorption efficiency increases with increase in shell thickness till 14 nm beyond which the Q_abs_ reduces (Fig. 2(b)) since the coupling between spherical and cavity plasmons gradually reduces at larger thicknesses. Meanwhile, Q_sca_ increases with increase in shell thickness and overrides Q_abs_ for larger shell thicknesses (above 25 nm). From Fig. 2(b) it can be seen that for Cu_2_O (50 nm) - Au core-shell particle with shell thickness 6 nm, plasmon resonance is at 1040 nm which is very close to the desired excitation of 1064 nm.

## FDTD Simulations On Cu_2_O (50 nm)-Au Core-Shell Particles

**Figure 3:**
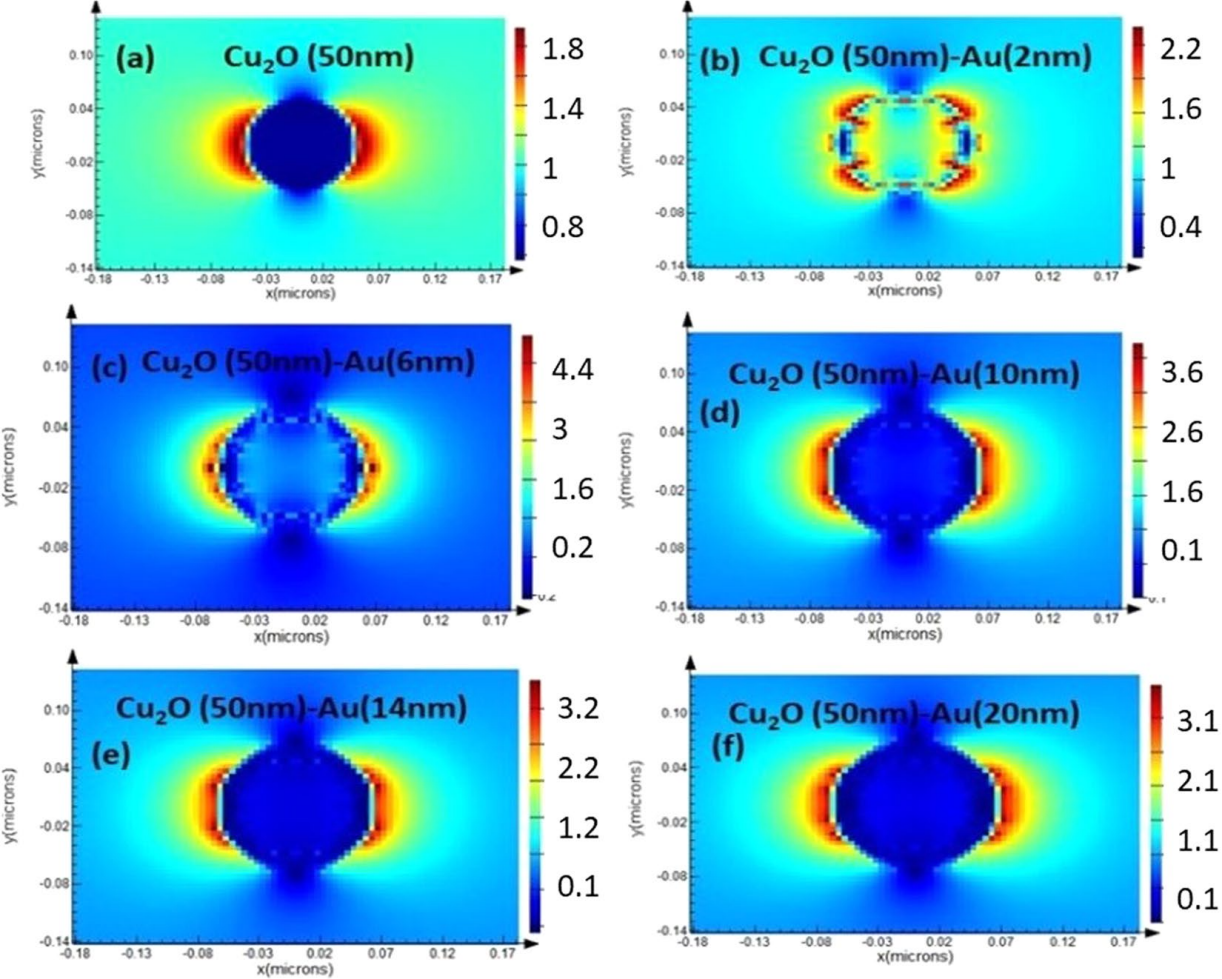
Electric field distribution (|E|) around Cu_2_O-Au core-shell particles at 1064 nm excitation for Au shell thickness 0 nm (**a**), 2 nm (**b**), 6 nm (**c**), 10 nm (**d**), 14 nm (**e**) and 20 nm (**f**).

FDTD simulations are performed using Lumerical software to study the electric field distribution over Cu_2_O (50 nm) - Au core-shell particle at 1064 nm (Fig. 3(a–f)). Simulations are performed by considering air as the surrounding medium. A gradual enhancement in the electric field around the particle with the increase in Au shell thickness is observed till 6 nm where the field intensity is maximum as the plasmon resonance at 6 nm shell thickness (1040 nm) is close to the excitation wavelength of 1064 nm. As the plasmon resonances move farther from the excitation wavelength, a gradual decrease in the near field is observed. We could also observe an abnormal field distribution in Fig. 3b.

In Fig. 3b, the thickness of Au shell is 2 nm (very close to the quantum regime) where the transverse motion of conduction electrons is quantized. Such quantum size effect results in the formation of two groups of electrons. One group provides non local contribution to the electromagnetic field response which can be explained classically and also can be studied using FDTD simulations. The other group executes quantum transition between states which are in resonance with the external wave. This can only be explained using the quantum approach and is not possible using FDTD which uses classical approach to solve Maxwell’s equations. Abnormal field distribution observed in Fig. 3b is hence due to the quantum size effect^15^.

## FDTD Simulations On NIR Absorption Enhancement of Cu_2_O (50 nm) - Au Particles

We have tried further to improve the NIR absorption efficiency by distributing Cu_2_O - Au core-shell particles on Au substrate using a graphene spacer layer. Graphene is chosen as spacer layer as it is a good absorber of NIR in combination with plasmonic nanostructures. It helps to effectively reduce reflections at substrate-particle interface and also renders efficient coupling of localized surface plasmons of core-shell particles with the Au substrate^31^. Graphene layer of thickness 1 nm (approximately three layers of graphene) is designed on Au substrate of thickness 50 nm.

**Figure 4:**
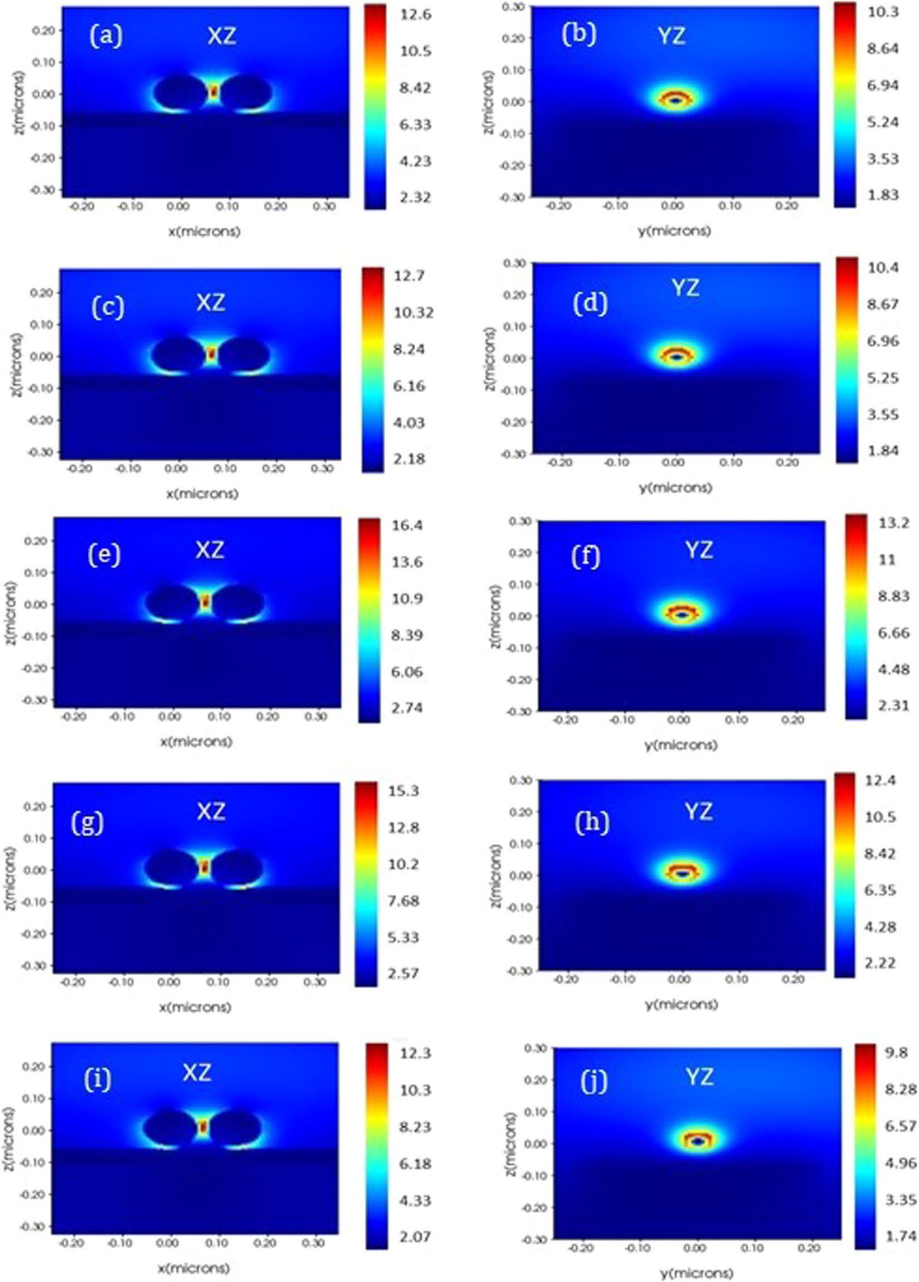
Electric field distribution around Cu_2_O - Au core-shell particles at varying thickness of graphene spacer layer. Figure 4(a,c,e,g,i) show field distribution in XZ direction and 4(b,d,f,h,j) in YZ direction with one, two, three, four and five layers of graphene spacer layers respectively.

Optimization of graphene spacer layer is done using FDTD simulations and the results are shown in Fig. 4. Simulations are performed with one to five layers of graphene as spacer layer. Electric field enhancement between two Cu_2_O (50 nm) - Au (6 nm) core-shell particles is studied in each case using two field monitors along XZ and YZ directions. Field intensity between the particles is observed to be the highest with three layers of graphene spacer which corresponds to an approximate thickness of 1 nm (Fig. 4(e,f)). Hence we have used the optimized thickness of 1 nm for graphene spacer in all simulations

**Figure 5:**
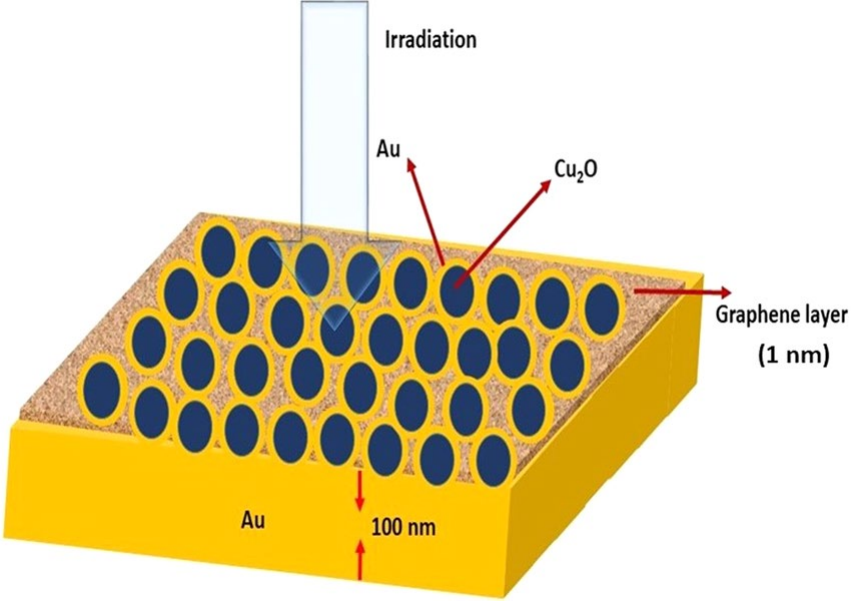
Schematic of (Cu_2_O - Au) – Graphene - Au layered structure.

Figure 5 shows the schematic of the proposed layered structure. Design of the structure and the study of its NIR absorption properties are performed using Lumerical FDTD solutions. Cu_2_O (50 nm) – Au core-shell particles are selected for designing the NIR absorber due to their large Q_abs_ values in the NIR region. Optimized Au shell is 6 nm for maximum absorption at 1064 nm for single Cu_2_O (50 nm) - Au core-shell particle. But the absorption scenario becomes entirely different when Cu_2_O - Au core-shell particles are distributed over the Au substrate. Plasmonic coupling between Au substrate and core-shell particles via graphene spacer layer significantly modifies the effective absorption by the designed structure. Hence the NIR absorption properties are further studied for different Au shell thickness values for layered structure. Single layer of Cu_2_O (50 nm) - Au particles are uniformly distributed over the graphene layer and the structure is then irradiated with NIR source (power = 1 mW). Two different power monitors are used to collect the transmitted and reflected signals from the structure when irradiated with plane wave source (λ = 0.7 µm to 2 µm). Perfectly matched layer (PML) boundary condition is employed in all simulations. The mesh size is taken as 0.2 nm which is well below the thickness of the spacer layer. The incident polarization is parallel to the substrate.

## Effect of Au Substrate On Plasmon Resonance of Cu_2_O (50 nm)-Au Particles

**Figure 6:**
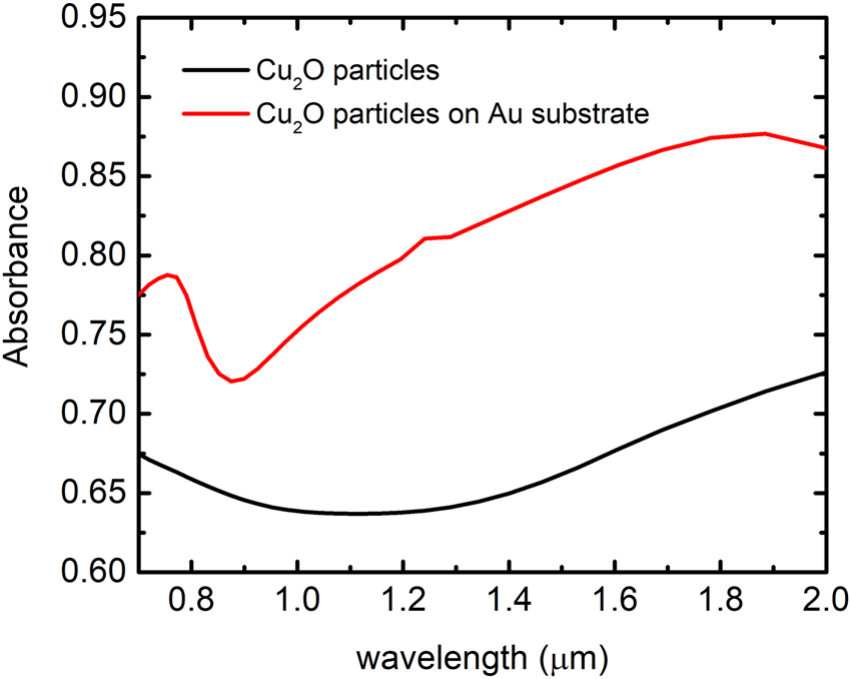
Absorbance of Cu_2_O particles in air (black) and Cu_2_O particles on Au substrate (red).

**Figure 7:**
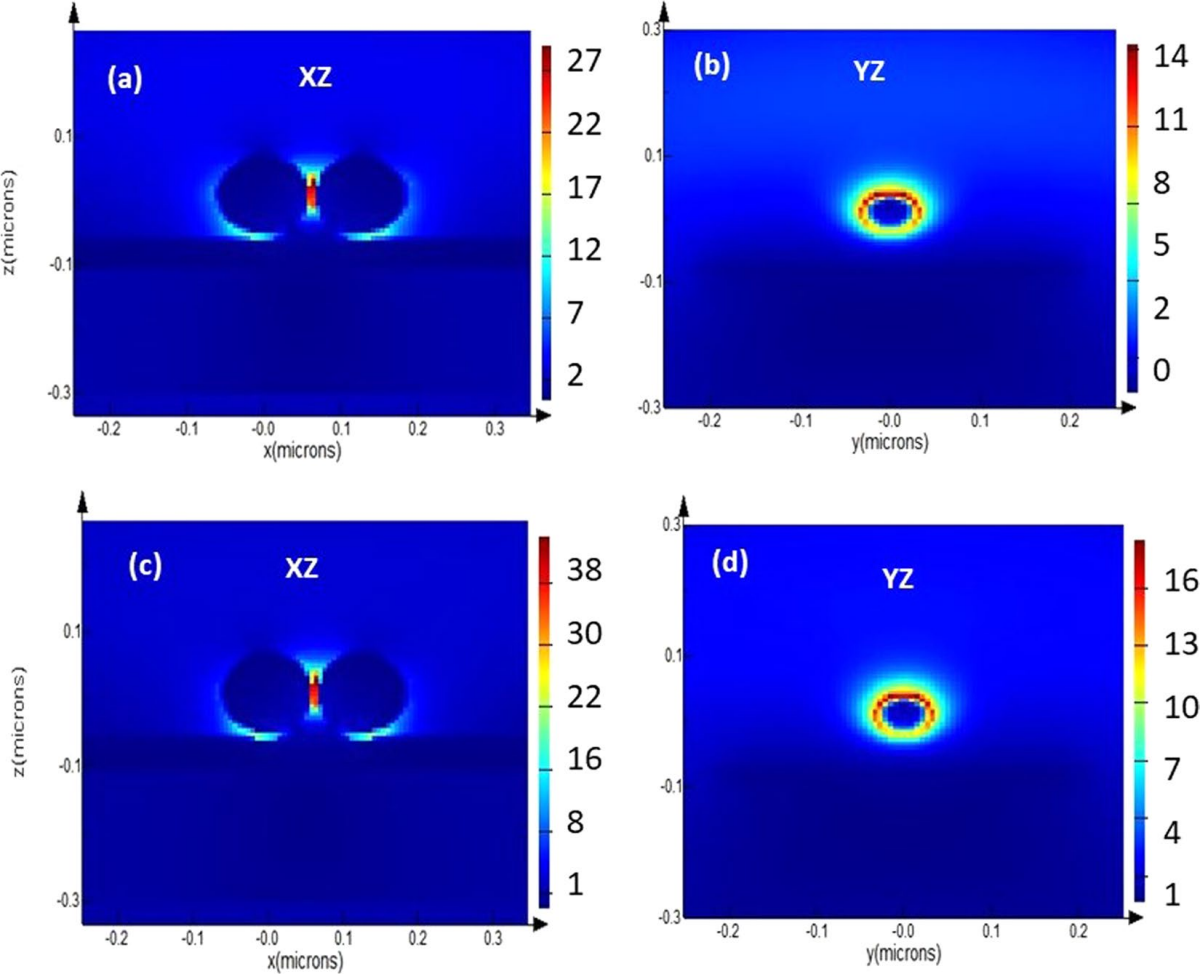
Electric field distribution of Cu_2_O (50 nm)- Au (6 nm) particles on Au substrate in the XZ plane (**a**) and YZ plane (**b**) and that of Cu_2_O (50 nm) - Au (6 nm) particles on Au substrate with 1 nm thick graphene spacer layer in the XZ plane (**c**) and YZ plane (**d**) at an excitation of 1064 nm.

In order to confirm the influence of Au substrate on NIR absorption, FDTD simulations are performed for two different configurations – Cu_2_O (50 nm) particles without Au substrate and Cu_2_O (50 nm) particles on Au substrate. Figure 6 shows absorbance of Cu_2_O (50 nm) in the presence and absence of Au substrate. Plasmonic field due to Au substrate is found to have a huge influence on the NIR absorption of Cu_2_O and is reflected as a clear enhancement in the NIR absorption over the entire NIR region. To prove the enhanced plasmonic coupling of core-shell particles with the substrate in the presence of spacer graphene layer, we have modelled two systems in which two Cu_2_O (50 nm) - Au (6 nm) particles are distributed on Au substrate with and without 1 nm thick spacer graphene layer. The excitation wavelength is chosen as 1064 nm in FDTD simulations. Field distribution in the XY and YZ directions without and with the spacer layer are shown in Fig. 7(a–d), respectively. Electric field between the particles is found to enhance by around 40%, and between particles and substrate by around 14% in the presence of graphene spacer layer thereby validating the stronger plasmonic coupling in this case compared to the bare Au substrate.

## NIR Absorption in (Cu_2_O (50 nm)-Au)-Graphene- Au Layered Structure

**Figure 8:**
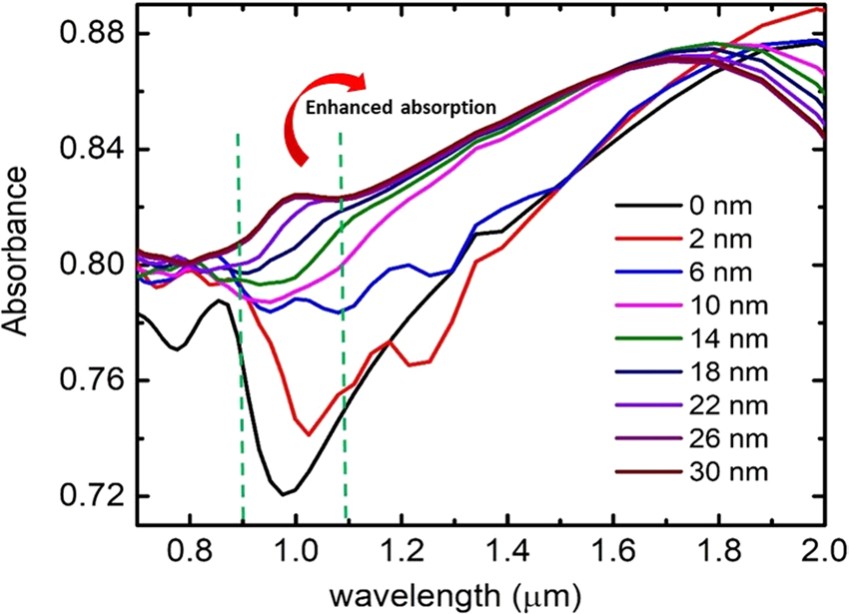
NIR absorbance of (Cu_2_O (50 nm) - Au) – graphene - Au layered structure for different Au shell thicknesses.

Further, FDTD simulations are performed on the layered structure shown in Fig. 5 by varying the thickness of the Au shell. Figure 8 shows the absorbance of the layered structure with Cu_2_O (50 nm) - Au core-shell particles with different Au shell thicknesses. Huge enhancement in absorbance is observed in the NIR region due to the plasmonic coupling between Cu_2_O - Au with Au substrate through graphene spacer layer. We could observe a gradual enhancement in NIR absorption over the entire range 0.7 µm to 1.6 µm when the Au shell is added which is the highest at 30 nm beyond which it is a constant. NIR absorption in the window 0.9 µm to 1.1 µm for (Cu_2_O (50 nm) - Au) – Graphene - Au layered structure increases by 18% compared to (Cu_2_O (50 nm)) – Graphene - Au layered structures. Increase in absorption in the specified window is attributed to the effective plasmonic coupling between Au substrate and core – shell particles. Local field around the core – shell particle has contribution from both the localized plasmonic field and the incident electric field. Enhancement in NIR absorption due to Au shell results in the formation of hot spots with stronger local electric field confinement between the core-shell particles. It is inferred from simulations that the hotspots provide significant enhancement factor values which are proportional to the fourth power of locally generated electric field. Generation of such strong hotspots enable SERS detection of biomolecules even with very low Raman cross sections. Our specific interest is in the region 0.9 µm to 1.1 µm where we could observe a new plasmonic peak originating at higher Au shell thicknesses due to the effective plasmonic coupling between the particles and the substrate. At lower Au shell thickness values, the dipolar and quadrapolar resonances of core-shell particles are far apart where dipolar resonances fall in the IR and quadrapolar resonances fall in the UV region. As the thickness of Au shell increases, quadrapolar resonances get red shifted towards visible region while blue shift occurs to dipolar resonances to visible region making both the resonances much closer. This results in the coupling between these modes causing enhanced effective plasmonic absorption over a wide range and formation of new plasmonic peaks. Such a strong plasmonic absorption within the NIR biological window is highly useful for sensing applications particularly sensing based on NIR surface enhanced Raman spectroscopy (SERS). The possibility of using (Cu_2_O - Au) – graphene - Au layered structures as NIR SERS substrate using FDTD simulations is studied further.

## FDTD Simulations on SERS activity of Cu_2_O-Au-Graphene-Au Multilayer Substrate

FDTD simulations are further used to study the scattering enhancement in Cu_2_O (50 nm) – Au particles when they are kept on Au substrate with graphene spacer layer. Scattering enhancement factor here is defined as (E/E_0_)^4^ where E is the maximum local electric field and E_0_ is the incident electric field. Gap between Cu_2_O (50 nm) - Au particles and also that between Au substrate and Cu_2_O (50 nm) - Au particles are maintained as 1 nm in all simulations. This enabled the plasmons from the particles as well as the substrate interfere constructively to produce highly enhanced local electric field regions within the gaps called hotspots. The excitation source of wavelength 1064 nm to study the NIR SERS property of the layered structure is Total - field scattered - field. It helps to separate the scattered field from the incident field and helps to avoid the reflections from the simulation boundaries.

**Figure 9:**
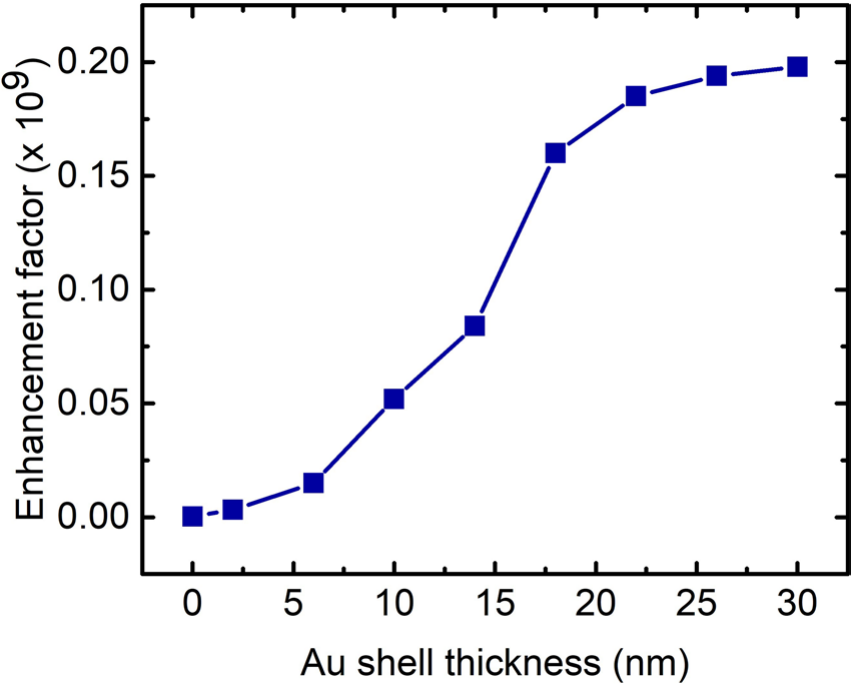
Electric field enhancement factors for (Cu_2_O (50 nm) - Au) – graphene - Au layered structure at different Au shell thicknesses.

Simulations are carried out for various Au shell thicknesses ranging from 2 nm to 30 nm and the scattering field enhancement factor, which is a measure of SERS activity of the structure, is calculated in each case. Fixed separation of 1 nm between the particles and that between particles and substrate is maintained in all simulations. SERS enhancement factor (EF) which is a measure of SERS efficiency of substrate is defined as (E/E_0_)^4^ where E is the local maximum electric field and E_0_ is the amplitude of input source electric field. With the addition of Au shell, EF is found to increase from 1.24 × 10^3^ to 1.86 × 10^8^ at larger shell thicknesses and the increase is observed to be very fast till the thickness value reaches 22 nm (Fig. 9). Beyond 22 nm, a slight increase in EF is observed giving rise to a stable order of magnitude 8 at 1064 nm excitation which points to the possibility of using the proposed structure for NIR SERS applications. Hence the proposed structure will serve as efficient NIR SERS substrate which in particular has applications in bio-sensing where most biomarkers such as fluorescein and AFP have their fluorescence in the visible region.

## Conclusion

We have proposed a novel metal - dielectric – metal layered NIR SERS substrate which can find immense potential in NIR based bio sensing applications. NIR SERS substrate with Cu_2_O - Au core shell particles distributed over the Au substrate with graphene spacer layer with optimized aspect ratio (Cu_2_O (50 nm) - Au (22 nm)) is found to show large electric field enhancement factor of the order of 10^8^ at 1064 nm excitation. Graphene spacer layer enables efficient plasmonic coupling between the upper layers of core-shell particles with Au substrate. Field enhancement factor values which are better than the reported NIR SERS substrates suggest the possibility of using the structure as efficient NIR SERS substrate for various applications. General drawbacks of visible SERS such as fluorescence background, photo bleaching and plasmonic heating which adversely affect the intensity of SERS signals can be overcome by the proposed structure as it is designed for a longer wavelength of 1064 nm.

## Methods

MieLab software is used for the study of plasmon resonance tunability in Cu_2_O – Au core – shell particles. MieLab is a computational package which simulates the scattering of electromagnetic field of multilayered spheres based on Yang’s algorithm^27^. Absorption efficiency (Q_abs_) values are calculated for various aspect ratios of Cu_2_O – Au core – shell particles towards their optimization for maximum absorption in the near Infra-red (NIR) region. Simulations are performed for three different Cu_2_O core radii −40 nm, 50 nm and 60 nm, respectively. Au shell thickness is varied from 2 nm to 30 nm in steps of 4 nm for each core radius value. Refractive index values of Cu_2_O and Au are provided as input parameter for simulations.

Finite difference time domain (FDTD) simulation using lumerical FDTD software is used for visualizing the electric field enhancement at the vicinity of Cu_2_O – Au core – shell particles. FDTD method is based on the classical solution of Maxwell’s curl equations. This method solves Maxwell’s equations on a discrete spatial and temporal grid (Yee cell)^32^. Perfectly matched layer (PML) boundary condition is used throughout the simulations. Plane wave source (λ = 0.7 to 2 µm) is used as the irradiation source. Simulations are performed at a mesh size of 0.2 nm. (Cu_2_O – Au)- Graphene – Au layered structure with optimized dimensional parameters are modelled as surface enhanced Raman scattering (SERS) substrate using lumerical FDTD module. Two frequency domain field monitors are used for collecting the transmitted and reflected signals when irradiated with the plane wave source. SERS enhancement factor (EF) is simulated for the optimized geometry of the substrate at an excitation wavelength of 1064 nm using FDTD simulations.
